# An Analysis of Android Malware Classification Services

**DOI:** 10.3390/s21165671

**Published:** 2021-08-23

**Authors:** Mohammed Rashed, Guillermo Suarez-Tangil

**Affiliations:** 1 Computer Science and Engineering Department, Universidad Carlos III de Madrid, Avda. de la Universidad 30, 28911 Leganés, Spain; 2 IMDEA Networks, Avda. del Mar Mediterraneo, 22, 28918 Leganes, Spain; guillermo.suarez-tangil@imdea.org

**Keywords:** Android, malware, classification, family, VirusTotal, antivirus, clustering, labels

## Abstract

The increasing number of Android malware forced antivirus (AV) companies to rely on automated classification techniques to determine the family and class of suspicious samples. The research community relies heavily on such labels to carry out prevalence studies of the threat ecosystem and to build datasets that are used to validate and benchmark novel detection and classification methods. In this work, we carry out an extensive study of the Android malware ecosystem by surveying white papers and reports from 6 key players in the industry, as well as 81 papers from 8 top security conferences, to understand how malware datasets are used by both. We, then, explore the limitations associated with the use of available malware classification services, namely VirusTotal (VT) engines, for determining the family of an Android sample. Using a dataset of 2.47 M Android malware samples, we find that the detection coverage of VT’s AVs is generally very low, that the percentage of samples flagged by any 2 AV engines does not go beyond 52%, and that common families between any pair of AV engines is at best 29%. We rely on clustering to determine the extent to which different AV engine pairs agree upon which samples belong to the same family (regardless of the actual family name) and find that there are discrepancies that can introduce noise in automatic label unification schemes. We also observe the usage of generic labels and inconsistencies within the labels of top AV engines, suggesting that their efforts are directed towards accurate detection rather than classification. Our results contribute to a better understanding of the limitations of using Android malware family labels as supplied by common AV engines.

## 1. Introduction

With more than 2.8B active users worldwide, Android is now the most used OS on mobile devices [1]. In a similar manner, Android has become the top target OS for smartphone malware. In the early days of the platform, between October 2010 and October 2012, Kaspersky reported an increase of incoming Android malware from less than 1 K to more than 40 K [2]. By March 2020, the influx of new malware reached 480 K [3]. Thus, since the beginnings of the platform, Antivirus companies (AVs hereafter) developed threat intelligence solutions to protect Android users from malware [4,5,6]. Because of the limited number of detected malware samples early on, human analysts were able to study samples, identify their behavior, and label them following an internal scheme of the AV company, most likely including the platform, type, and family of the sample (see Section 5.2). However, such a surge made it inevitable for AVs to use automation techniques in both detection and family classification because of the impossibility of manually handling the influx of samples arriving to AVs [7]. Gheorghescu, a researcher at Microsoft’s Security unit (as indicated in the affiliation), introduced his automatic family classification system and indicated, in 2005, that his technique was not generally adopted by the industry [8]. We note that Microsoft^®^, though not an AV company itself, is a major key player in the AV industry [9]. The research community has long relied on labeled datasets to carry out studies on the prevalence and evolution of Android malware, or to validate novel detection and classification methods. While the authors of the Android Malware Genome Project’s dataset (Malgenome hereafter)—possibly the earliest academic Android malware dataset—analyzed each sample manually to identify its behaviors and families [10], manual analysis is the exception today in this area. Most works in this area (e.g., [11,12,13,14,15,16,17,18,19,20,21,22,23]) have relied on VirusTotal (VT) to build their datasets by using the labels that VT engines gave to each sample. It is well known, however, that AVs tend to focus their resources towards accurate detection rather than classification. This implies that many suspicious samples are labeled with generic labels rather than with more identifying ones. As a result, the class and family of the sample are often vague, as we show in Section 5.3. In addition, as each AV has its own methodology, determining if a given sample belongs to a certain class or family might differ from one AV to another. While these issues apply to all forms of malware, they are especially acute in the case of Android. That is because there is less expertise in the industry with regard to Android malware due to the novelty of the platform, unlike desktop malware which has been studied for decades by both the industry and the scientific community.

To build large labeled datasets, many researchers rely on a two-step process: (1) scanning samples/hashes on VT, and (2) using tools for unifying labels of samples, such as AVCLASS [24] and Euphony [18]. While this process might not seem problematic at first glance, using such tools can very easily introduce noise in the dataset building process silently if the underlying AV engines do not provide coherent family labels. The problem lies in that 23% of security research works relied on AV results as ground truth for either malware detection or malware family classification [25]. It is only natural to assume that this reliance extends to Android security related works. On a related note, Perdisci et al. [26] addressed the flaw of using majority voting, which label unifying tools, such as AVCLASS, rely on, and proposed an alternative technique so as to establish a better ground-truth that does not discard the non-selected labels of majority voting. The so-called “consensus” between AVs in label unifying tools gives a false sense to the researcher of an established ground-truth for their dataset, even though such consensus only covered 20% of the samples, as reported by Bayer et al. [27]. Such homogenous datasets would not represent the distribution of malware seen in the wild.

In this paper, we shed light on the limitations associated with using VT’s malware classification services to determine the family of Android malware samples. Knowing these limitations is critical for researchers when building a labeled dataset as it will act as their ground truth/reference when analyzing the threat landscape or when training classifiers. Our study shows that researchers heavily rely on VT labels in creating their Android malware datasets and demonstrates that each of the AV industry’s big players that we studied (almost all are in the list of VT engines) has a limited view of the threat ecosystem, which leads to label discrepancies amongst them. Indeed, we are able to confirm this cause of label discrepancies by showing the high disagreement level between the VT engines from the results of our measurement experiments, which show a maximum of 64% of single AV coverage (how many samples an engine flags as non-benign) and 52% for AV pair coverage. In a similar fashion, we find that the best agreement level between AV pairs for samples whose family name could be identified by AVCLASS2 [28] does not go beyond 29%. Overall, our study aims at helping researchers understand the limitations of using VT along with label unification tools in Android malware classification, and taking additional precautions when using them instead of relying on them blindly. Our contributions are summarized in the following:

- We study the historical malware family classification methods, as well as those of Android. We compare the latter to the industry’s practice of Android malware classification.
- We perform an exhaustive study of the industry’s Android malware labeling phenomenon between 2012 and 2020 by analyzing reports of several AV vendors. We do the same in academia by studying relevant papers from top security conferences between 2011 and 2020. To the best of our knowledge, we are the first to do so.
- We analyze 2.47 M Android malware samples using VT and calculate the coverage of single AVs, as well as between each AV pair of the top 10 AVs in coverage.
- We study the prevalence of the usage of generic labels and uncover several inconsistencies within the labels of each of the top 10 AVs in coverage.
- Using the Rand index metric, we determine the level of agreement on family and class between each AV pair in the top 10 AVs.

The rest of the paper is organized as follows: In Section 2, we provide background on malware family classification methods, as well as the industry practices in this context. In Section 3, we study how both the industry and the research community use malware family labels. To do so, we analyze reports from several AV companies, as well as the 8 top security conferences in the period of 2012–2020 and 2010–2020, respectively. Then, in Section 4, we introduce the dataset we use for the measurement and describe our coverage analysis. Section 5 addresses the labeling problem, its challenges, and our findings when scanning our dataset on VT. Section 6 explains our experiments that measured family and class coherence across different AVs. Section 7 provides constructive discussion on the key takeaways of our work, our recommendations, and the limitations. Finally, we discuss related work in Section 8, while Section 9 concludes the paper and provides details on possible future work.

## 2. Android Malware Classification

This section provides necessary background on the problem of classifying malware into families, with emphasis on approaches—both academic proposals and industry-based systems—that target samples for the Android platform.

### 2.1. Malware Classification

The problem of classifying malware into families has traditionally been approached from a statistical learning [29], as well as other perspectives, using both unsupervised and supervised learning methods. Unsupervised Learning is used with unlabeled datasets, where no previous knowledge of the sample’s family exists. On the other hand, Supervised Learning requires a labeled dataset, which is split into training and testing sets.

We next provide examples of highly cited works that focused on *Unsupervised Learning, Supervised Learning*, or both together. We do not consider the use of *Unsupervised Learning* in dimensionality reduction as use of both together but, rather, only take into account the usage of both in determining the family.

#### 2.1.1. Unsupervised Learning

While clustering is not the only existing approach in Unsupervised Learning [30], it is used extensively in the context of malware analysis. For instance, Bayer et al. [27] proposed a clustering approach for Portable Executable (PE) malware based on a scalable dynamic analysis. The system which leveraged data tainting to identify dependencies between system calls was capable of analyzing 75 K samples in less than 3 h.

Bailey et al. [31] built a behavioral clustering-based classification system that executes samples in a sandbox and extracts the fingerprint from system state changes as they are more invariant than abstract code sequences. They compared their results to several AVs to identify the limitations of the AVs. AVs at that time mainly used signature-based techniques, which surely failed against unseen/new malware.

With the goal of having an automatic classification approach to help analysts deal with the growing number of malware samples, Kinable and Kostakis [32] introduced a system based on Call Graph (CG) clustering. Representing malicious samples as CG allows identifying structural similarity while allowing some degree of variation. They used Graph Edit Distance (GED) to calculate the distance between any two CGs; CGs were based on local and external functions. They then used the distance to identify if a sample belonged to any cluster.

#### 2.1.2. Supervised Learning

Ahmadi et al. [33] designed an efficient a Machine Learning (ML) system which extracts complementary statistical features related to the structure of PE files. These features are extracted from both the hex dump and disassembled code and include information, such as the file size, entropy statistical measurements, and histograms of strings and frequencies of: (a) opcodes, (b) common APIs, (c) special characters, (d) keywords, and (e) register use. The small size of the set of features allows for the system’s scalability. The system is able to identify the family even for packed samples. It was tested on Microsoft’s Malware Challenge dataset [34] (https://www.kaggle.com/c/malware-classification accessed on 15 June 2021) and yielded an accuracy of 99.8%.

Kolosnjaji et al. [35] designed a deep learning system that modeled system call sequences based on two parts: Convolutional and Recurrent. The Convolutional part has convolution and pooling layers. The convolution layer’s goal is extracting features from one-hot vectors, while the pooling aims at dimensionality reduction. This part does not model the sequences of system calls but, rather, counts their existence, as well as the relation of n-grams. Simplifying sequence modeling leads to losing information fidelity. On the other hand, the output from the previous part are connected to the recurrent part; where each output is represented as a vector. The recurrent layer uses Long-Short Term Memory cells to model the resulting sequence, which enables modeling the sequential dependencies of the API calls. The authors then used pooling to identify the most important features and reduce complexity. To avoid overfitting, they depended on Dropout. The last layer they used was a softmax layer that generates label probabilities.

Kalash et al. used deep learning, a branch of ML, for classifying samples into families from two datasets of 10 K and 22 K samples [36]. They converted samples into images which they then compared to the training set for each family and assigned the sample under examination to the family that had the highest matching score.

Hu et al. [37] built a KNN-based system that uses call graph matching with the aim of creating an industry-like scalable system that receives massive number of samples daily. To overcome the time-consuming graph matching, they relied on approximate GEDs, as well as early pruning, in the graph tree.

Gheorghescu [8] designed an ML system that could run on average desktop machines. His system, which relies on features extracted from Control Flow Graphs (CFG), compares unseen samples with previously seen malware, which is stored in a database (DB), and would return matches based on existing malware’s evolutionary behaviour. He tested three algorithms: ubiquitous edit distance (ED), B-Tree structure, and Bloom Filters (BF), on his system and compared their accuracy and execution time. While ED was more accurate than B-Tree and BF, which had identical results, BF showed much faster query and insertion time in the DB, as well as less storage space.

Nataraj et al. [38] used visual similarity as a basis for malware family classification. They adopted a KNN-based approach that requires no execution or disassembly of the sample. The system reads samples as a vector of 8 bit unsigned integers then organizes them into a 2D array (image representation) with fixed width and varying height based on the file size. The execution speed makes the system scalable, although it would fail against samples packed with different packers that belong to the same family.

Tian et al. [39] base their classification system on strings extracted from samples disassembled via IDA [40]. Unexpectedly, many strings used in the classification originate from library code, which means that library code is not distinguished/excluded from the program’s disassembled code before the string extraction process. The authors compared the performance of several algorithms, such as KNN and AdaBoost, and their system reaches an accuracy ranging between 91% and 97%.

#### 2.1.3. Hybrid (Supervised + Unsupervised Learning)

Rieck et al. [29] introduced an incremental analysis family classification system that integrates both supervised and unsupervised learning. Their system, which is based on behavioral reports resulting from dynamic analysis, matches behavior to previously identified malware families and uses clustering for unseen ones.

### 2.2. Classifying Android Malware

Malware classification academic research is quite primitive in Android and, thus, is an area that needs more rigorous exploration and study. We highlight some of the used techniques in this area below.

**Static vs. Dynamic Analysis**. Generally speaking, family classification systems may rely on static [11,13,14,41,42], dynamic [43], and even hybrid (static + dynamic) [44] analysis of the samples. Static analysis involves the analysis of the sample without executing it. The systems that we mentioned used features from: (1) The dex code: API calls, Information Flows, Control Flow Graph, Call Graphs, (2) native code, (3) resources, (4) package metadata, (5) certificate, and (6) the manifest: components, intents, and permissions. The main advantages of static analysis is its lightweight. However, for malware that uses obfuscation, indicators of maliciousness may not be detected at all. On the other hand, dynamic analysis requires executing the sample in a sandbox, which generates a report with details on the read/write operations, network connections, sent/received sms messages, and input/output system calls; features are extracted from those reports. While having its advantage because of the transparency of code obfuscation in its environment, many newer versions of malware detect the execution environment and would not execute their malicious code inside a sandbox. Dynamic analysis, to the contrary of the static one, is quite costly in terms of time and resources. Thus, much research is focused on the lower cost option. On the other hand, the hybrid technique mixes the advantages of both types of analysis while still being costly. However, hybrid systems could be used in a more intelligent manner by using dynamic analysis only when the system fails to detect maliciousness using the static one.

**Feature Selection**. Both the static and dynamic features are extracted using known tools in the community, such as Androguard [45] and apkTool [46], for static features, and Droidbox [47] and Cuckoo [48] for dynamic ones. Yet, these tools usually generate lots of features and sometimes a feature selection algorithm is needed to discard the less important ones.

**Classifier**. With regard to using classifiers to study Android malware, we note that:

1. *Supervised Learning*: requires a labeled dataset, which is split into training/testing sets. The designer chooses how to split the set. We have seen 50/50 training% versus testing% splits [42,44], 80/20 [43,44], and 90/10 [11,41], as well as 66.6/33.3 [13,14]. Used algorithms include KNN [42], SVM [14,43], and Random Forest [44].
2. *Unsupervised Learning*: Clustering is mainly used. It requires no labeled datasets and consequently no dataset splitting. One common algorithm, which was used in Reference [44], is DBScan.

**Metrics**. Authors of the Android malware classification systems that we studied measured the quality of their systems using certain indicators/metrics. These metrics included True Positive (TP), True Negative (TN), False Positive (FP), False Negative (FN), and True Positive Rate (TPR), among others, that derive from these essential ones.

### 2.3. Industry Practices

The AV industry, while focusing more on detection as we show later, aims at classifying Potentially Harmful Apps (PHA) into families, as well. We note that we are only able to provide very limited details on the industry practices due to the lack of transparency from AV companies; we have rigorously looked into white papers and reports by AV companies and were able to obtain generic rather than more focused information. We found that, generally, AVs integrate several techniques in their family classification systems. These methods include:

1. Signature: is the most classical method used for classifying already known malware where the AV matches the malicious code of the sample in question to a fingerprint of malicious code in its repository [49,50].
2. Heuristics: includes static and dynamic heuristic analysis. The static heuristic analysis compares the decompiled code of the sample to suspicious code in previously found malware and flags the samples as a possible threat if the suspicious code passes a certain threshold. The dynamic one executes the sample in a sandbox and flags the sample if suspicious behavior is found [51].
3. Machine Learning: where samples are collected from different possible sources, such as shared threat platforms, honeypots, end-users, etc. AVs may use supervised, as well as unsupervised, ML techniques to create their model, on which they classify samples into distinct families [9,52,53,54,55].

We have seen over the past few years how different AV companies have proliferated while relying on third-party engines. This responds to a classical business strategy where some companies focus on commercializing technology that they outsource to others by developing state-of-the-art methods in the field. For example, *BitDefender*^®^ and *Avira*^®^ are two known outsourcing engines. According to Reference [56]: (1) Emsisoft^®^, Tencent^®^ and G- Data^®^ use *BitDefender*; (2) F-Secure^®^ uses *Avira*; and (3) Qihoo 360^®^ uses both *BitDefender* and *Avira*.

Yet, even though AV vendors share threats and samples [9,55,57,58,59], they are still competitors and try all means to find the adequate model to maximize their profit.

## 3. Usage of Android Malware Family Labels

In this section, we demonstrate how both the AV industry and the academic research community use Android malware family labels. We discuss the lack of consistency across AV vendors, issues in transparency about the methodologies used, and the noticeable effects of the challenges behind curating a dataset.

### 3.1. Usage by the AV Industry

We first analyze malware reports from several AV vendors, as well as Google, to identify prevailing families.

**Methodology.** We searched for reports from prominent companies published since the emergence of Android OS. First, we searched on Google using keywords and key phrases, such as “threat reports”, “cyber threat intelligence reports”, “top malware families in Android”, etc. Then, we manually went through all the reports and annotated the aliases of different malware families. Next, we matched the annotated aliases against known threat encyclopedias (https://www.microsoft.com/en-us/wdsi/threats, accessed on 10 October 2020) (https://www.fortiguard.com/encyclopedia, accessed on 10 October 2020), which showed varying results for labels (more on this in Section 5.2). We also studied reports by government agencies and used VirusTotal to ascertain that our annotations related to family names.

**Results.** In total, we include information from 30 reports from 6 companies (Nokia, Sophos, CheckPoint, Google, Kaspersky, and Symantec) ranging from 2012 to 2020. We only retain reports from companies that discuss Android malware families specifically. Table 1 provides the total number of reports/year that we retain and use in our study. We note here that we gathered more reports from additional years, as well as from more AVs, but we discarded them due to the lack of information on Android malware families.

We find that the reports are predominately marketing-oriented, and they generally lack consistency, even for the same entity, be it: (1) in the frequency of the reports (e.g., Nokia’s reports for the 2nd half of 2014, 1st and 2nd half of 2015, and all-year for 2017, 2018, and 2020), or (2) in the format of the report, where they lack information regarding families in one year and provide it in others (e.g., Google provides generic information mixed with examples of families in 2016’s report and includes more family names in other years).

We also observe that some AV reports are oriented towards different threat components of the malware ecosystem. This is only natural as each vendor prevails in certain geographical markets more than others. Thus, AVs (unlike Google) do not have a global situational awareness and provide different versions of the most significant threats. Besides, the inconsistency in malware naming and the use of different aliases for the same family adds complexity to the analysis, possibly making readers believe that different aliases represent different threats.

To understand these differences, we analyze the set of common families between the AVs. Table 2 summarizes the top most popular families found. We emphasize the challenge we encounter when analyzing the commonalities between vendors due to the lack of consistency in the naming convention. We find that, even though Google and some AVs intersect in some families, sometimes, it is difficult to uncover this intersection. Google’s family naming tend to be more flashy; they use animal names (e.g., FlashingPuma, SnowFox) and food (e.g., IcicleGum, BreadSMS). On the other hand, AVs use more threat-oriented names (e.g., Opfake, HiddenApp). We attribute this to the markets which each of them targets. Google’s choice of names follows a long line of dessert names for its OS releases, while the AVs’ choice is based on showing their (potential) customers the effectiveness of their solutions in combating threats. We highlight the AVs’ inconsistency in reporting the number of prevailing families: Kaspersky reports the top 20, Symantec reports the top 10, and Google does not tend to provide a fixed number of top threats. Likewise, AVs provide percentages of threats, sometimes vaguely through figures (e.g., Sophos), while Google provides none.

**Takeaways.** We extract these takeaways from our analysis:

- Companies are not consistent in (a) including family names in their reports and (b) providing detailed technical information about these families. Instead, reports are rather generic with a shallow technical details and geared towards showing the effectiveness of their solutions.
- Lack of consensus between AVs on the most prominent threats of each year. Even in years when there are a large number of reports discussing specific families (i.e., 2016–2018), consensus is limited; the peak occurred in 2018 with **Triada**, where 4 out of 6 vendors included it in their top families’ list.
- Even as time passes, transparency in reporting families does not seem to improve, which confirms their marketing tendency versus the technical one: only 2 vendors published reports in 2019 and 2020, although we had 5 reporting vendors in 2018.

### 3.2. Usage in Academic Research

We next study how the research community leverages Android malware labels for different measurement studies, classifier design papers, or others.

**Methodology.** We performed a semi-manual analysis of Android related papers published in top computer security conferences, between 2011 and 2020. In particular, we looked at: (1) CCS, (2) USENIX Security, (3) S&P (Oakland), (4) NDSS, (5) ACSAC, (6) RAID, (7) ASIA CCS, and (8) DIMVA. We then went through the Web pages of each conference year by year, whenever available, and searched for a set of keywords in the title and abstract. If the Web page of the conference in that particular year was not available or did not list the abstract, we checked the proceedings of the conference. We used the following keywords in our search: *malware, android, google, mobile, app, dataset, data set, smartphone*. Finally, we qualitatively studied all resulting papers.

**Results.** We identified a total of 81 papers that use an Android malware dataset; we refer the reader to the paper distribution in Appendix A in Table A1. We clarify that there are papers in other areas of the Android research space that receive the attention of researchers but are not relevant to our study—papers looking at developer’s methodology or the study of 3rd party libraries are a few examples. Table 3 shows the prevalence of datasets used in the literature. We group together papers according to the dataset they use. In the table, even though we use the **UNK** in the lower bound whenever we lack information about the number of samples, it is more of a formatting matter, and it might perfectly be the upper bound. We note that:

- Several papers use datasets that they craft from AV companies. Although each one of them is unique, we aggregated them into a single row called *Companies* for better readability.
- *Custom* refers to non-specific datasets that were built by the authors themselves for their work and were not released to the community.
- *Unknown* are those sets for which we could not identify if they were *Custom* or samples from a different repository/dataset.

We also report the number of samples in each dataset. The largest dataset has about 4.6 M samples, but the most popular datasets are several orders of magnitude less. When a dataset is distributed with labels, we report the number of families. Families range from 49 to 179 and labels usually come from VT after being harmonized (see Table 3, column Class. Mthd). Finally, we account for collection period as reported in the paper describing the dataset. Only a few papers detail the collection period. Regardless of the collection period reported, Table 4 uses VT to show the first time the samples in a dataset have been seen in the wild (namely, *first_seen*).

Another phenomenon we came across is the aggregation of several datasets that come from different sources such as the case of Zhang et al. [136]. While this helps researchers to obtain more samples, it can change the representativeness of real world scenarios when combined with live feeds, such as those given by VirusShare. We believe such representativeness is exclusive to AVs and that academic solutions will stay in the proof of concept area. We address this more in the next section.

Finally, Table 5 indicates the different objectives that our set of papers have. Papers may have more than one purpose, which explains that the sum of the # Papers is more than the total number of papers we surveyed. We also note the following:    

1. **Detection** does not necessarily mean malware detection only but, rather, any type of detection (e.g., Reference [16] focuses on ransomware detection).
2. **Analysis** indicates any analytical/measurement study, e.g., comparison between families, benign and malware, results from AVs, etc.
3. **Family Classification** also includes type classification, e.g., ransomware versus other malware types.
4. **Tools** whose goal is not detection/family classification, such as forensic tools, tools for protection, etc.
5. **Other** refers to purposes not falling in the above categories. In our set of papers, this includes attack design, fingerprinting apps through traffic, model-checking based detection, and others.

**Takeaways.** Based on our findings, we deduce the following:

1. *Lack of ground truth.* Most datasets do not contain family labels. Understandably, only a few early and small datasets were the result of manual labeling efforts, e.g., Malgenome [10]. Knowing the morphology of a dataset (i.e., the family labels) may inform practitioners of how the dataset is balanced towards certain type of threats. While this might have a lesser impact on malware detection research, the lack of ground truth for the families is an obstacle for those studying malware family classification.
2. *Use of VirusTotal (VT).* Academic researchers rely heavily on VT to analyze (and label) their samples: 18 out of the 32 datasets in Table 3 had their samples scanned on VT in at least one of the papers that used that dataset. Researchers generally label a sample as malicious if it is flagged by more than a certain threshold VT engines, e.g., Reference [94], ignoring the fact that many AV products rely on 3rd party AV engines [56].
  For the research community, the dilemma lies in that VT is an easily accessible resource. However, we argue that this resource should be used with a good understanding of its limitations. For instance, VT relies on a simplified version of the represented AV engines https://blog.virustotal.com/2012/08/av-comparative-analyses-marketing-and.html (accessed on 5 December 2020, which introduces limitations when seeking consensus or comparing engines.
3. *Unknown representativeness.* We find information about when the samples are generally collected. However, details about when the samples were observed in the wild and their subsequent prevalence are usually not reported. We observe discrepancies across papers regarding such dates, even in known datasets, such as Malgenome [10] and Drebin [12]. For instance, the authors of Reference [12] rely on VT to obtain their labels; they mention that the dataset contains samples seen between from 2010 and 2012. However, when we independently query VT, we find that the *first_seen* dates are between 2009 and 2013 (see Table 4). Taking into account sample dates is important to avoid: (a) training classifiers with outdated samples that are no longer representative of the threat landscape or (b) training the classifier with recent samples while testing it using older ones. However, obtaining an accurate value of the date in which a sample has been in operation can be challenging. First, threat intelligence might not be available. Next, timestamps from the samples can be easily tampered with. Finally, estimating it from the min. SDK and the max. SDK offers a very coarse range of dates.
4. *Label harmonization.* It is a rather common practice to use semi-automated processes to perform label harmonization when performing family classification. A popular tool is AVCLASS [24], although there are others, e.g. Euphony [18] and AVCLASS2 [28]. (Euphony and AVCLASS2 were used in Zhang et al. [136] and Sebastian and Caballero [28] consecutively. We do not include them in Table 3 because they were not used when crafting a dataset but, rather, when authors were designing their experiments.) Manual labeling was used only in Reference [10] when authors inspected the set manually to determine the family of each sample; something not scalable when constructing bigger sets.
5. *Use distribution.* Out of 146 uses of the datasets in our study, more than 70% use 7 popular datasets. Malgenome alone is almost used 25% of the time, even in recent works [28]. On the other hand, our analysis shows that about 30% of the works use an unpopular set. Some of these sets are proprietary or unavailable. This is a clear sign that authors have confidence in well established datasets. For instance, Wang et al.’s dataset [113], which appeared in 2018, has 4.5 M samples and is accessible to the community but was only used once.

## 4. Coverage Analysis

To take a deeper look at the AV labeling techniques used by the industry, we build an extensive dataset of over 2 million samples. We next present an overview of our dataset, and then we evaluate the coverage that different engines have over it.

### 4.1. Our Dataset

To build a comprehensive Android malware dataset, we rely on several sources. We then query VT to collect intelligence from the AVs.

**Data sources.** One of our main sources is AndroZoo [96]. At the time of writing, AndroZoo contains a little more than 13 M apps collected from 14 different markets and repositories, including Google Play. For each sample, AndroZoo provides the number of VT detections, as well as the sample’s hash (sample hereafter). We filter apps with 1 or more detections, which leads to a set of 2.55 M apps that we query on VT to obtain the AV labels. When querying VT, we found that about 300 K samples were no longer considered malware by any of the VT engines. Consequently, we only retain the remaining 2.24 M samples that have at least one AV detection at the data collection time.

Besides, we collect samples from academic datasets and commonly used repositories. We complement that with samples from the OTX AlienVault platform [137] and from Palo Alto Networks [138]. Table 3 shows a summary of all the data sources and the number of samples. After removing duplicates, our final set includes approximately 2.47 M samples, for which we provide more details, as well as the distribution of the samples over the years 2009–2020, in Table 6.

**Dataset Characterization.** After analyzing the intelligence offered by VT, we discovered that the number of AV detections vary from sample to sample. For example, some AVs report samples as undetected, possibly white-listed because the samples are known to be “goodware”. These very AVs do not appear at all in other sample reports, which might be because they refrain from making a decision in these cases because the sample was namely *unseen*. We encounter a total of 100 different AV engines in all the reports collectively. This is different than the number of engines reported in other works; a few examples are 56 engines in both Arp et al. [12] and Wei et al. [94], 60 in Salem et al. [139], 65 in Zhu et al. [140], and 77 in Dashevskyi et al. [19]. In our dataset, we found the average number of engines per report to be 58.7 AVs, the minimum of AVs per report to be 13, and the maximum 68. VT’s behavior with regard to having an unfixed number of scanners for each report is orthogonal to the problem we are studying, but in the scope of our future work.

Another observation we have is that some AV vendors contribute with more than one engine, for example, *Sophos* versus *SophosML*, and *TrendMicro* versus *TrendMicro-housecall*, to name a few. Additionally, 5 engines have 0 detections for the entire set of samples (i.e., they report all samples in our dataset as undetected or unknown), 9 with less than 10 detections, 17 less than 100 detections, and 33 engines with less than 1K detections; the latter representing 0.04% of the dataset. Thus, from an original set of 100 engines, after discarding all these low detection engines, we are left with with only 62 engines to look into.

**Figure 1:**
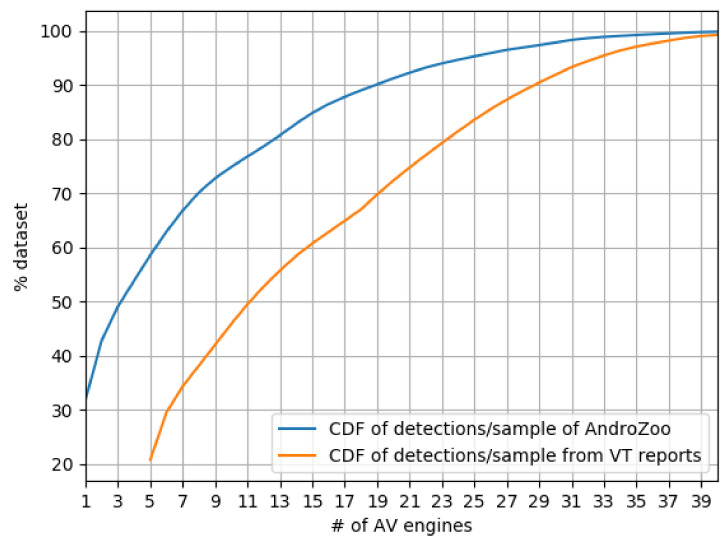
CDF of AV detections for samples in our dataset. x-axis represents # of engines detecting samples; y-axis is the percentage of samples in dataset detected by this # of engines.

Figure 1 summarizes the prevalence of AVs that mark samples in our set as malware based on the VT reports. We compare this with the number of detections that AndroZoo recorded from VT previously (Note that other datasets do not report the first seen; thus, our comparison is restricted to AndroZoo). We conclude that there is a general disagreement between AVs on whether a sample is malware or not. To detect 90% of the samples, collective results from 29 engines were aggregated (cf. CDF from VT reports). However, with just 10 additional engines on the same curve, the CDF reaches almost 100% of sample detection.

Furthermore, we find that the same set of samples have more detections over time as we compare our snapshot of VT reports with AndroZoo’s, while others disappear,particularly samples in the 1–5 band. We note how AV engines “flip” their decision from detected to undetected, and vice versa. Overall, about 13% of the samples that have 1 detection as per AndroZoo, have 0 hits in our latest snapshot. We do not investigate further the reasons of these detection flippings, but we note that our observations go in line with related work looking into the flipping decisions of AV engines over time  [140].

**Takeaway.** Our findings suggest that Android malware detection is a highly specialized task within the ecosystem. Only a few engines offer comprehensive detection. In particular, we find that the top 10 AVs jointly flag at least 85% of the dataset.

We, thus, focus the rest of our analysis on the top 10 most effective AV engines according to their coverage, as defined next.

### 4.2. Results of Coverage Experiments

**Figure 2:**
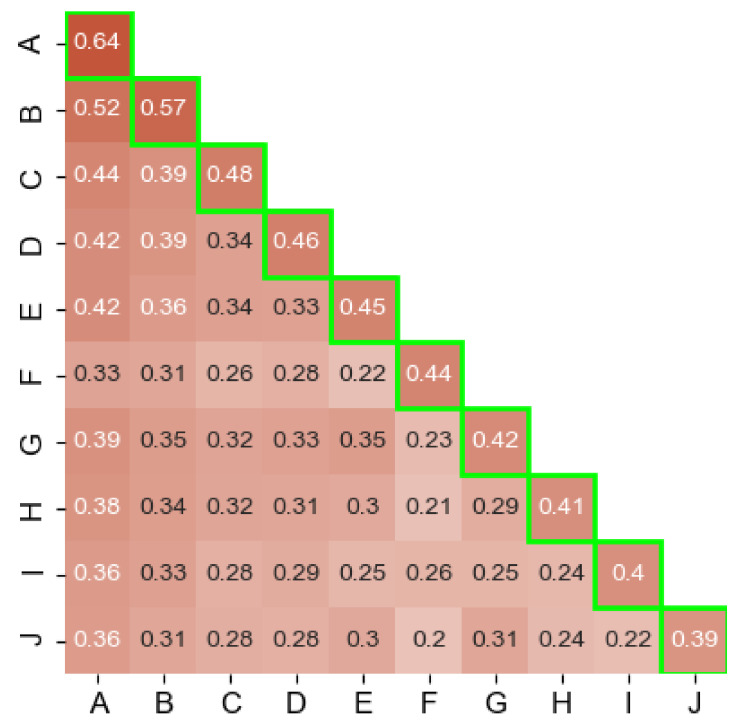
Heatmap for the top 10 engines in coverage. (**A**): eset-nod32, (**B**): ikarus, (**C**): fortinet, (**D**): cat-quickheal, (**E**): nano-antivirus, (**F**): symantecmobileinsight, (**G**): avira, (**H**): cyren, (**I**): k7gw, (**J**): F-secure. The cells with the light green edges are those that represent the single coverage for the engines.

**Single AV Coverage.** We first analyze the fraction of samples that have been detected as malware by at least one engine (which changed to 5 after rescanning the samples) and are given a non-empty label. We refer to them as labeled samples hereafter. Our results are summarized in the highlighted main diagonal of Figure 2. Even though the top 10 AVs collectively account for a large portion detections, the individual engine coverage only ranges between 39% and 64%. This shows that the performance of the AVs drop considerably when it comes to reporting actionable intelligence in the form of labels. We note that these results do not necessarily indicate label correctness. To get a deeper understanding of this issue, we later measure the consistency of the labeling process.

**Coverage across AV engines.** Besides, we measure the coverage across every AV engine pair (cross coverage hereafter). To do so, we count the number of labeled samples that are flagged by both AVs. Given that we focus our analysis on 10 AVs, this results in 45 possible AV pair combinations. The values of the cross coverage are shown under the main diagonal in Figure 2.

In general, our results show that the cross coverage is predominantly lower than the single coverage of most of the 10 engines. The average cross coverage is 32%; much lower than the 46% average of the single coverage. We note that the largest overlaps are with **ESET-NOD32** (labeled as A in Figure 2), with the highest value being 52% for the *(ESET-NOD32, Ikarus)* pair and the lowest overlap’s value being 20% in the case of the *(SymantecMobileInsight, F-Secure)* pair.

When we compare both the cross coverage and single coverage in Figure 2, we observe the proportion of samples that 2 engines share and that they do not. For instance, A has labels for 90% (52/57) of B’s samples. On the other hand, B alone has labels for 5% (57–52) of the entire dataset and, in turn, A alone has 12% (64–52). The 10 engines collectively agree on just 6% of the samples. In addition, we find that the samples that are flagged solely by any of the top 10 AVs, along with AVs outside of the top 10 (there are at least 5 flags/sample), represent only 15% of our dataset.

## 5. Label Analysis

We next discuss the original convention of “malware” labels that was introduced in the early 1990s and how it is compared to real-world labels assigned by AVs today. We address the fact that the real-world labels are inconsistent, causing problems for researchers who then have to seek help from label unification tools that we describe as well. Additionally, we discuss the inconsistencies that we discovered when analyzing our dataset.

### 5.1. Labeling Convention

In addition to detecting malware, AV engines generally provide a label that characterizes the malware specimen detected. There have been various attempts by the industry to standardize the label format. In 1991, the Computer AV Research Organization (CARO) [141] suggested the following naming convention:

*The full name of a virus consists of up to four parts, desimited by points (‘.’). Any part may be missing, but at least one must be present. The general format is Family_Name.Group_Name.Major_Variant.Minor_Variant[[:Modifier]*,

Where *Family_Name* represents the family that characterizes the virus; *Group_Name* represents similar viruses within the family (i.e., sub-family), and *Major_Variant* and *Minor_Variant* are modifiers used for structural and behavioral differences across variants.

The key principle behind the CARO naming scheme is that malware samples can be grouped into families according to their code similarity. Even though CARO was never fully adopted, the majority of AV engines today use labeling schemes that follow the same principles, going from the more general information to the more specific. In addition to the components described above, labels are often prepended with tags describing the platform and the malware type. The platform refers to the execution environment of the sample, which includes Operating Systems, frameworks and scripting languages. The type refers to the main threat category in terms of malicious behavior, such as Trojan, Worm, Ransomware, Virus, Backdoor, etc.

### 5.2. Labeling Challenges

While AVs adhere generally to the CARO convention, some use labels that are very generic or indicate the use of a heuristic to characterize the sample. Others use labels lacking a taxonomy or a naming convention but, rather, represent technology or are just generic labels. For example, McAfee often uses the term *Artemis!<some automated string>*, where *Artemis* is the name that the company gave to the technology it uses to systematically characterize samples [142]. The first challenge lies in that the naming convention used to describe labels is heterogeneous, and there is a lack of a proper taxonomy.

AV label unification tools, such as in References [18,24,28], are an important step forward, where AVCLASS2 [28] is the most recent one. It is a platform agnostic tool that obtains the common labels from VT reports that have non-empty labels from 2 or more engines. It extracts 5 categories: 1. Behavior (BEH): captures the malware behavior when the samples was executed, e.g., *spam, sendsms*, 2. CLASS: equivalent to CARO’s type, 3. Family (FAM): malware grouping based on similar characteristics, such as having the same author [143], e.g., *Lotoor, Triada*, 4. FILE: equivalent to platform described in Section 5.1, and 5. **UNK**: a category for concepts which are neither in the taxonomy nor in the tagging rules. AVCLASS2 could generate one or more UNK tags. The tool discards any generic labels, e.g., *Trojan*. The authors of AVCLASS2 used millions of labels to identify categories that are real ones compared to those categories that add no value. Additionally, the tool has an update module which allows extending the original so as to include new instances in the four main categories. Similarly, the authors of Reference [18] have studied the different taxonomies of labels but just for Android. Based on their findings, they created lexing rules on which they divide a label into four categories: (a) type of threat, e.g., virus, worm, (b) platform: same as the one defined in Section 5.1, (c) family name, and (d) information: extra description of threat, e.g., variant. Because of the lack of knowledge, changes in labeling methods, etc., the authors propose a set of 10 heuristic rules so as to map AV label tokens to one of the categories, where it also relies on a database of initial knowledge on malware labels.

The first challenge is faced when using such label unification tools as that requires fine-tuning; an error-prone task. For instance, it requires considerable domain knowledge to understand that *Artemis* is McAfee’s a generic term. Yet, pre-defining this term is a critical step to avoid subtle errors during label unification. Another related challenge is that the labeling methodology is unknown and not consistent across vendors. This has significant implications when drawing the line between variants and families, which is a challenging task, as shown in Reference [144]. However, AV vendors make such judgment on a regular basis, which results in different levels of granularity (across vendors). For example, in spite of **Microsoft**’s engine on VT considering *FakePlayer* and *FakeBrows* different families, we find that **Dr. Web** considers both as variants of the same one *Android.SmsSend.2* and *Android.SmsSend.401*, respectively [145,146]. This inconsistency in the ground-truth can mislead approaches based on supervised-learning. In fact, a recent work showed that reviewing labels is of paramount importance so as to be able to perform reliable automated classification [147].

Third, some AVs may be using a third-party AV engine for labeling, as already explained in Section 2.3. This requires further consideration when using thresholds to decide on a sample’s maliciousness or label. Finally, given the fact that vendors share samples (see Section 2.1), some approaches involve looking at these relationships and taking them into account. Approaches, such as that used in Kantchelian et al.  [148], weigh AV labels based on performance metrics and consider correlations that can negatively impact the confidence (weight) given to a vendor.

### 5.3. Engines Label Findings

To understand better our set, we did a visual-manual analysis for the label taxonomies of the top 10 engines in coverage. Generally speaking, except for **SymantecMobileInsight** and **K7GW**, the other AVs lack consistency in their label taxonomy. Due to limited space, we highlight the most interesting findings for each AV.

1. **ESET-NOD32**: 3 K samples have the label *multiple detections*. Sometimes the string includes *a variant of* before the label and sometimes it includes *potentially unwanted* or *potentially unsafe* after the label. 193 K samples, i.e., 12% of the AV’s flagged samples are labeled *a variant of Android/Packed.Jiagu.<some_variant> potentially unsafe*. Yet, Jiagu refers to Jiagu 360, a famous Chinese packer, not a family name.
2. **Ikarus**: We found labels, such as *AdWare.Adware*, *AdWare.ANDR*, and *AdWare.AndroidOS*. We are not able to explain the inconsistency in these similar labels. In addition, samples whose labels lacked a family name but used the word *Qihoo* and *Jiangu* (both chinese packers) are quite frequent.
3. **Fortinet**: Lack of class name is quite frequent, e.g., *Android/Styricka.A!tr*. *Riskware/Jiagu!Android* is the top label with 103 K samples (9%). *Android/Agent.FS!tr.dldr* represents 4% of the labeled samples. *Adware/Waps!Android* and *Adware/Waps.G* appear to be the same, but one has the platform name, while the other lacks it (they collectively represents 4.5% of labeled samples).
4. **Cat-QuickHeal**: Lack of identifiable family name, e.g., in *Android.km.Aeb23 (PUP)* is *km* the family name or just a randomly generated string with some automation tool? Generic labels *Android.Downloader.N* and *Android.Agent.GEN23333 (PUP)* represent 8% of the dataset. Similarly, *Android.Jiagu.A (PUP)* represents 4.3% of the set.
5. **Nano-Antivirus**: 5% of the samples are labeled *Riskware.Android.Agent.entxvm*, which is the top label in the set.
6. **SymantecMobileInsight**: 64% of the labels are not associated with a family. The labels follow this format: *AppRisk:Generisk*, *AdLibrary:Generisk*, *Other:Android.Reputation.1*, and *Other:Android.Reputation.2*
7. **Avira**: 4.7% of the samples have the generic label *ANDROID/Dldr.Agent.FS.Gen*, the second most frequent label. Labels with numbers as family name, e.g., 33 in *Android/DownLoader.33.31*, are not uncommon. In addition, we are not sure if the family name can be represented by all uppercase or the family part is just randomly generated and the samples lacks a family name, e.g., *AMAA* in the label *ADWARE/ANDR.AdDisplay.AMAA.Gen*.
8. **Cyren**: It uses completely different taxonomies in its labels, such as *AndroidOS/GenPua.95BB2BF2! Olympus* and *Trojan.BYLR-9*. In addition, there is a lack of family name in the first, and unidentifiable family name and missing platform in the second.
9. **K7GW**: Lacking family name, as well as platform, in all of its labels, e.g., *Adware (0053361f1)*, *Exploit (004c0f451)*.
10. **F-Secure**: Lack of consistency in top labels *Adware:Android/AdWo* and *Android.Adware. Adwo.A*, which represent, collectively, 8.4% of the labels.

We deduce from this analysis that AVs generally are unable to identify the family names in a clear and definite manner. There maybe several reasons for that:

- Prioritizing detection over family identification. This is seen in **ESET-NOD32**, **Fortinet**, and **Cat-QuickHeal**, which give packed samples labels related to packing rather than their unwanted behavior.
- Lack of knowledge of the territory because of Android’s relative novelty compared to the well-established expertise in Windows, which has been there for decades.

The lack of a globally agreed on taxonomy between vendors is a source of confusion to researchers who use labels in their studies. Even single vendors have several taxonomies, which makes label analysis even more difficult. Based on our analysis of labels, as well as the philosophy on which CARO bases its taxonomy, we suggest one that goes from the upper level structure to the lower level details. We use the same categories used by AVCLASS2 and Euphony that we explained earlier. Thus, our proposed format would be as follows:

*Platform(File).Class(Type).Behavior.Family.FamilyVariants*.

We note that one of the main difficulties we faced when looking at labels from the different AV engines was the variation in the number of levels, i.e., some labels lacked some of the categories in our recommended taxonomy. Hence, to facilitate label analysis and study, labels need to have some kind of globally agreed on null character in those categories whenever the value is undecided.

## 6. Family and Class Analysis

We next report results on our analysis of the family and class labels assigned by each AV engine to detected samples. We focus on:

- Measuring the ability of unifying tools (AVCLASS2 in this case) to extract family and class names from the samples that each of the engines flags. We also run the same experiments for the AV pairs.
- Measuring the coherence across engine pairs when grouping together samples according to their families by identifying the agreement level among the family clusters of the different AV engines without taking into consideration the actual family names used by the engines. We repeat the analysis for the class labels. Both coherence experiments help us determine if the underlying classification methodologies used by different AVs produce similar results or not.

### 6.1. Family and Class Name Extraction

**Figure 3:**
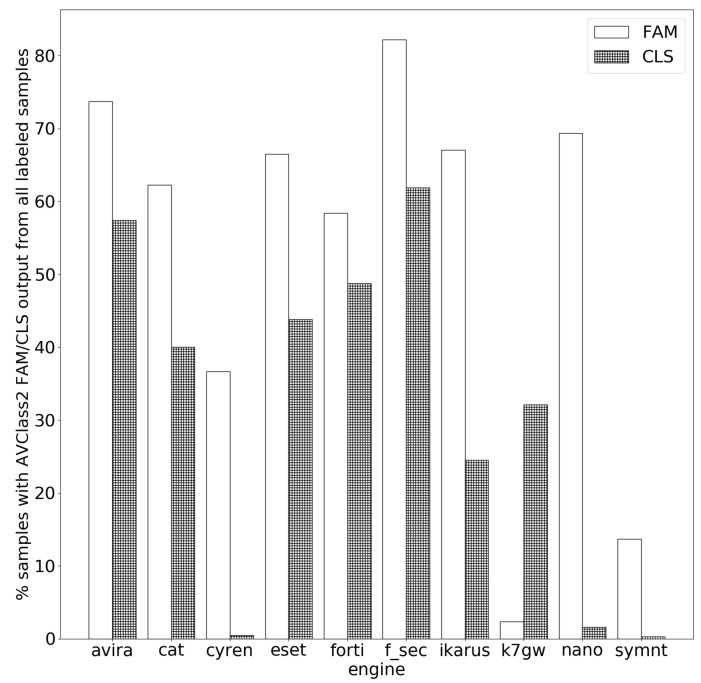
Percentage of family/class detections in all labeled samples for the top 10 engines in coverage.

**Extracting family names for single AV engines.** We rely on AVCLASS2 to extract family names from the labels. AVCLASS2 generally requires at least two engines to extract common categories. Thus, we build our own scripts on top of it so as to extract the family and class, even from a single AV engine. The percentage of family name extraction with respect to the total number of labeled samples for each engine is shown in Figure 3. We observe that the ratio of samples for which we successfully obtain a family label ranges between 2% and 82% (with respect to labeled samples not the entire set), with the majority of values falling in the 50–70% range (average is 53%). This indicates: (a) samples are not labeled with clear extractable names, which is probably the main reasons that leads to (b) AVCLASS2’s failure to detect the family name from a substantial amount of the labels. Unclear labels are caused by the engine’s failure to attribute the sample to a particular family. One of the reasons behind that is perhaps the use of heuristic detection techniques that indicate abnormal or malicious behavior without attributing it to a particular family. Examples include **SymantecMobileInsight**’s label *trojan:genheur*, **Ikarus** labels *not-a-virus:HEUR.RiskTool.AndroidOS.Agent*, *HEUR.Trojan-Spy.AndroidOS.Agent*, *HEUR.Backdoor.AndroidOS*, and *HEUR.Trojan-SMS.AndroidOS.Tiny* and Avira’s labels *ANDROID/SMSSpy.HEUR.Gen*, and *ANDROID/SMSHEUR.C.Gen*.

**Extracting class names for single AV engines.** We repeat the previous experiment for the class name. The results are shown in Figure 3, as well. Except for **Nano-Antivirus**, we notice a clear drop in the percentages of class name extraction compared to the family’s. We attribute this change to two factors: (a) some engines use labels that lack classes but have a family name (e.g., **ESET-NOD32**’s label *a variant of Android/Anserver.E* has the family name *Anserver* but no class name); (b) inconsistency between label formats, which makes it impossible for AVCLASS2 to parse them because of the engine’s taxonomy that does not take the category into account. This is clear in the case of **SymantecMobileInsight**, whose label taxonomy’s main pattern is *Behavior:Family*, causing AVCLASS2 to detect only a small subset of the classes.

**Figure 4:**
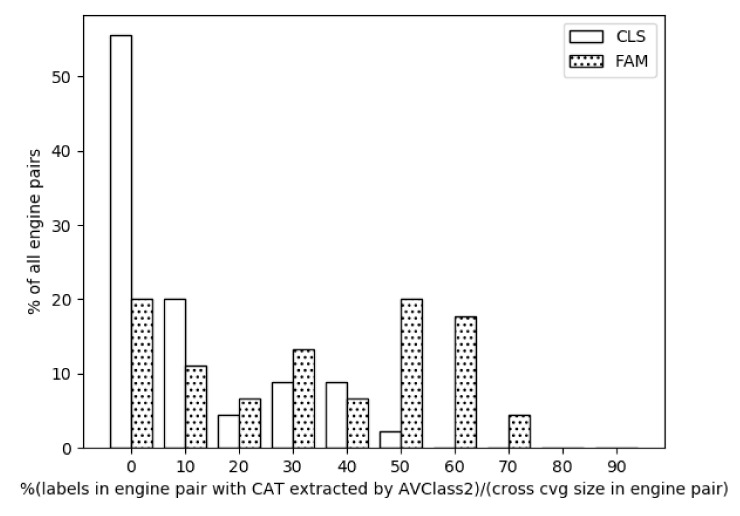
Histogram for $\frac{FAM/CLASS\;label\;identification\;in\;both\;engines}{total\;cross\;coverage\;for\;engine\;pairs}$ after using AVCLASS2 (***A2***) to extract FAM/CLASS. max(***A2*** FAM) = 77%, max(***A2*** CLASS) = 55%. **CAT** here refers to **category** and not Cat-QuickHeal.

**Extracting family names for AV engine pairs.** We carry out this experiment by using results from the single family names experiment. For this, we count the samples for which families were obtained for both engines, regardless of whether they hold the same name or not. The results are seen in the histogram shown in Figure 4, which has 10 bins. The ratio of family name extractions by AVCLASS2 to the size of cross coverage ranges from almost 0% up to 74%, where i≤bini<i+10 for i=0,1,…,9. We found that the most frequent bins are those of 0%, 50%, and 60%, each representing around 20% of all the AV pairs. This is much different than the values of the single AVs, which were concentrated in the 50–70% range. In addition, the mean was found to be 37%, a significant drop compared to the 53% mean for single AVs. We attribute this to the fact that the performance of each engine is different than the others.

**Extracting class names for AV engine pairs.** We repeat the experiment of the family pairs but this time for the class pairs. Similar to the drop in single class name extraction compared to the single family name extraction, the pair class name extraction is notably lower than its family counterpart as seen in Figure 4.

### 6.2. Classification Coherence across Services

Given the lack of knowledge about the internals of the AV engines and their labeling methodology, we explore how similarly these engines classify malware samples into the same clusters. To this end, we base our analysis on a well-known metric used to compare the output of clustering algorithms: the Rand index [149]. According to Warrens et al. [150], *“the Rand index [...] may be interpreted as the ratio of the number of object pairs placed together in a cluster in each of the two partitions and the number of object pairs assigned to different clusters in both partitions, relative to the total number of object pairs. Thus, the Rand index combines two sources of information, object pairs put together, and object pairs assigned to different clusters, in both partitions.”*

In our context, we use the Rand index as follows. For every pair of AV engines $AV_{a}$ and $AV_{b}$, we compute the dataset $D_{a,b}$ for which both AV engines produce family labels. Let $N_{a,b}=\left|D_{a,b}\right|$ be the size of such dataset. Let $A=\{a_{1},\ldots ,a_{x}\}$ and $B=\{b_{1},\ldots ,b_{y}\}$ be the clusterings of $D_{a,b}$ into families according to $AV_{a}$ and $AV_{b}$, respectively. That is, each $a_{i}$ is the group of all samples in $D_{a,b}$ that $AV_{a}$ labels with the same family name. The same applies for $b_{j}$ with respect to $AV_{b}$. We now compute the next two quantities: *x* is the number of pairs of sample labels in $D_{a,b}$ that are in a certain cluster in *A* and in its corresponding cluster in *B*. Similarly, *y* is the number of pairs of sample labels in $D_{a,b}$ that are in different clusters in *A* and in different clusters in *B*. Intuitively, *x* and *y* measure the number of agreements and disagreements in the labelings of *A* and *B*, consecutively. The rand index for these labelings is computed by normalizing the sum over all possible matchings:

R(A,B)=x+yN2.

The Rand index allows us to measure the similarity between the family labeling done by two AV engines, regardless of the actual family names given by each product. Its value ranges between 0 (denoting full disagreement) and 1 (meaning that both engines produce the same assignment). To compute each of the $D_{a,b}$ datasets for each pair of AV engines, we rely on results from Section 6.1 for family and class name extractions for AV pairs. When applied to the family names, we refer to the result as the *family coherence* for the pair of AV engines. In the same manner, when the objective is to measure the class clusters, we call the output *class coherence*.

### 6.3. Family and Class Coherence

**Family Coherence**. We carry out 2 tests, which we explain below, to measure family coherence. We call the engines in each AV pair *AV_1_* and *AV_2_*.

1. We use the results of the **Family Name Extraction** experiment of AV pairs as an input.
2. We complement the list of family names with full labels of *AV_i_,* for which AVCLASS2 could not obtain a **FAM** value.

**Figure 5:**
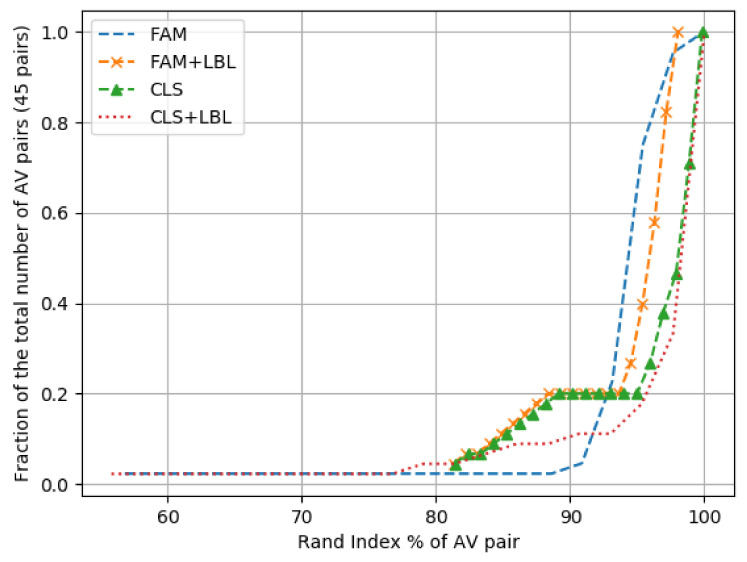
FAM/CLS Commonality: AVCLASS2 detects common Family (FAM curve) or common Class (CLS curve) in labels from both engines. FAM/CLS + LBL: AVCLASS2 families are complemented with the complete label if no FAM/CLS can be detected in both engines. AV engines Rand_Index values are lower whenever labels (LBL) are included.

We provide the coherence results for family only, as well as family complemented with full labels, for samples that do not have a family name in the curves FAM and FAM+LBL, consecutively, in Figure 5. While our experiment above has its own limitations, it is still a best effort because of our inability to obtain the family name from every sample. The limitations are in part due to the fact that many samples have either unclear family names or do not have a family name at all. We have seen this phenomenon in the majority of engines and provide one example only from **Avira** for the sake of brevity: (a) *ANDROID/AdDisplay.Zdtad.G*, (b) *ANDROID/AdDisplay.3443048*, and (c) *ADWARE/ANDR.AdDisplay.AMAA.Gen*. In (a), we can clearly identify *Zdtad* as the family, but (b) has only non-family categories, and we are not sure about (c) having a family or just randomly automated characters, especially given that they are all in uppercase. We realized that labels that mix uppercase and lowercase were clear family names, but those having only uppercase seemed to be just automatically generated strings. We leave the study of this phenomenon for future work. In addition, another limitation was because of the inconsistency in the taxonomies of the engines. With the exception of **SymantecMobileInsight** and **K7GW**, all the engines had no clear consistency in their taxonomies. This made our task difficult when it came to clearly identifying the family name on our own. Thus, we decided to use the full label whenever **FAM** was not available, as explained earlier.

**Class Coherence**. We repeat the coherence experiment for the class level. We first only use the samples whose class was extracted for both AV. Afterwards, we complement the experiment with the full labels of the samples whose class was not extracted. The class coherence results for the CLASS only experiment, as well as the CLASS label, that is complemented with the original label, for samples that lack it are demonstrated in the CLS and CLS + LBL curves, consecutively, in Figure 5.

### 6.4. Analysis of Family and Class Coherence Results

We realize a notable change after including full labels when measuring coherence for each of FAM and CLASS. The inclusion of full labels leads to a drop in the coherence values of both, which is clearly seen in Figure 5 when comparing the curves FAM and FAM+LBL for the family coherence case, and the curves CLS and CLS + LBL for class coherence. This means that the family/class clusters are negatively affected when including full labels; an expected consequence of the inclusion of these less regulated tags (the full labels). It also indicates the lower commonality in these tags as compared to the FAM/CLASS tags.

Yet, it is of essence to note that the coherence results when including full labels require much further study. That is because of our findings regarding AV engines’ usage of several taxonomies in their labels (Section 5.3). Another factor related to the previous one is the existence of several generic labels which could be reduced to just a single label for each AV. We refrained from unifying these labels due to the unclear labeling taxonomy that the AVs use as we mentioned earlier. We limited our work to the comparison of full labels in their original state because we lacked the knowledge of how each AV engine’s labeling methodology is.

While AVCLASS2 is a great tool for identifying families (as well as other categories), it is limited to a certain set of families in its dictionary. Even with its expansion module and the **UNK** pseudo-category which can help identify certain tags that are not in the tool’s taxonomy nor the tagging rules, such knowledge is quite limited for the research community when it comes to closed-source tools, such as the AV engines, as we mentioned earlier in Section 2.1. An extensive knowledge of each engine’s set of tagging rules in addition to the families it uses is a requirement to be able to add such rules to AVCLASS2’s expansion module. Only in that case can a researcher rely blindly on a preconfigured label unifying tool in identifying family labels for custom-built datasets. Additionally, the high Rand Index scores seen in the FAM curve in Figure 5 suggests that, whenever AVCLASS2 can identify the family for both engines, there is a great similarity in the family clusters between engines AV1 and AV2. This indicates that, whenever the industry key players have a clear agreement on a family, they tend to to classify its samples similarly. It also means that, in case a family is identified by both engines, family unification using tools, such as AVCLASS2, is possible. The same conclusion applies to the classes as seen in the CLS curve in the same figure, although it has slightly lower Rand Index scores. On the other hand, we saw how the Rand Index dropped in the curves FAM + LBL and CLS + LBL of the same figure whenever the family/class could not be identified in any of the engines or both. This shows that the use of AVCLASS2 or other similar tools could have its unseen impact on the ML algorithm when designing an ML-based family classifier.

## 7. Discussion

Our study shows that current AV engines are unable to provide meaningful family/class labels for a great portion of Android malware samples. This is reflected in many of the generic/vague labels, as we showed in Section 5.2. We are able to confirm this conclusion, based on the results of our experiments, which showed the low percentage of family name extraction in the case of single coverage (cf. Figure 3), which is even lower for cross coverage (cf. Figure 4).

This could be due to the vendor’s limited view, as explained in Section 3.1. Even though there is an ongoing collaboration between vendors, we believe that they mainly focus their efforts towards detecting ongoing threats to their users based on the markets in which they are active. Although Google has a bird-eye view on the Android malware status, which could help in improving AV engines’ results, we failed to identify any involvement of Google in the main threat collaboration platforms [9,55,57,58,59]. The only collaboration platform it is involved in the App Defense Alliance [151], which also includes ESET^®^, Lookout^®^, and Zimperium^®^. Out of these 3 companies, only ESET^®^ is on VT. Such an alliance allows these 3 to use Google Play Protect to obtain and share samples. In fact, this might be the reason why **ESET-NOD32** has the best coverage in our set.

On the other hand, AVCLASS2’s limitation might be partially because of its Android FAM/CLASS dictionary. While it includes an update module that allows for including unknown tags, we did not use it because of the impracticality of analyzing all the labels of the dataset. We have seen cases of using label unifying tools, particularly AVCLASS, blindly to build datasets [23,113], and we believe that this practice should be avoided by our community for several reasons. First, we already know from Section 2.3 that some AVs use the engines of other AVs. In addition, Zhu et al. [140] discussed how some AVs influence others in their flagging decisions. Thus, it would be wise that these factors are taken into consideration and that AVs that rely on 3rd party engines, as seen in Reference [56], are treated as one, along with those external engine.

Based on our analysis, we confirm the general strong disagreement between AV engines with regard to the Android malware ecosystem. We base that claim on two observations. First, our AV industry’s report analysis uncovered the lack of consensus on top families per year, as shown in Table 2. We note that, despite the scarce details that vendors provide, we consider those reports as literature-based indicators about the absent consensus. Second, we corroborate the previous observation with the results of our experiments in Section 4, Section 5 and Section 6. For instance, the average flagging rate for single AVs, as seen in the diagonal of Figure 2, is 46%, which drops to 31% for the cross flagging case between AV pairs. Furthermore, in Section 5.2, we provided evidence of how some engines consider samples to be variants of the same family, while others consider them as belonging to several ones. All of these observations prove the extremely limited agreement between AVs. This only means that these engines are generally weak with regard to Android malware detection and consequently weaker with classification.

While the labels that our set of engines uses obey CARO’s naming convention, the CARO convention itself is outdated. AV vendors, who are already collaborating on threat intelligence (see Section 2.1), need to work together on designing a clear and well defined labeling convention. A new labeling convention, even for generic labels, will help researchers to dissect, study, analyze, and understand PHAs with more ease, something that will come to the benefit of the AV vendors because their focus is geared towards detection. We confirmed our conclusion of the AVs’ focus on detection rather than classification after contacting a representative of one of the AV vendors. He told us that, although his company’s analysts try their best to get accurate detection labels, the real objective is to protect the users, which might eventually lead to misclassifications.

Additionally, in the curves FAM and CLS in Figure 5, we demonstrated how the coherence results for families as well as classes were generally quite high among the engine pairs when FAM, as well as CLASS, were identifiable in both of the AV pair engines. This shows that vendors agree on well-defined threats. Yet, we cannot disregard the fact that the percentage of the samples whose family was obtained laid between 2% in the case of **K7GW** and 82% in the case of **F-Secure**, with four out of the 10 engines having a FAM extraction rate in the 60% bin, as seen in Figure 3. This is even worse in the case of CLASS, whose values laid between almost 0% in the case of both **SymantecMobileInsight** and **Cyren** and 61% in the case of **F-Secure**; 3 out of the 10 engines laid in the 40% bin. These values, for both FAM and CLASS, surely prove that engines tend to give generic, less deterministic labels and focus their efforts more towards detection.

Moreover, given our knowledge that **F-Secure** relies on **Avira**’s core engine [56], we found a difference of 8% in the coverage, as seen in Figure 2. Yet, this percentage rises up to 10% for family names and drops to 4% with regard to class names (cf. Figure 3). We expected the percentages to be closer to 0% rather than oscillating between 4% and 10%. We deduce that, even though vendors with **rebranded engines** use engines of other vendors, they might have their own contributions towards the **rebranded engines** to improve their own engine’s performance. Another possible reason might be simply that the engine versions that each of these AVs use on VT are not the same.

### 7.1. Recommendations

The fact that AV pairs have much lower cross coverage average than single coverage average is a strong indicator of the infeasibility of blind label unification. This idea becomes more substantiated after taking into account that the best AV pair family intersection *(ESET-NOD32,Ikarus)* represents just 29% of the dataset, with this pair having the highest value in cross coverage. The family intersection values are almost evenly distributed among the *0, 10, 20 bins* with the lowest value being 0.07% *(SymantecMobileInsight,K7GW)*. With all these findings in mind, we advise the community to take into account the following when building Android malware datasets:

- Understanding the interdependencies among the different engines and discarding results from redundant ones, such as in the case of **F-Secure** and **Avira.**
- Taking into account: (a) the tendency of AV engines to give generic labels instead of family identifying ones and b) the variability of generic labels, e.g., **Avira**’s generic labels: *ANDROID/Agent.qzqlq, SPR/ANDR.Agent.cdhey, Android/Spy.Agent.FI.Gen*.
- Machine-Learning-based family classification systems that discard samples with generic labels, despite their very high accuracy, would not stand in a real-world scenario where there is non-stop influx of incoming malware. In these systems, the classifier is fitted with features extracted from the samples in the dataset and would be greatly affected by changes to the structure of that particular dataset. ML have been historically tied to ground-truth non-changing samples, such as image recognition, and applying that to a continuous changing field, such as malware detection/classification is challenging [7]. In addition, these classification systems cannot be compared to AV engines, whose goal is detection rather than family classification.
- Blind usage of label unification tools does not take into account the variability in family names that AVs might use for the same family, thus introducing noise into the ML algorithm. For example, samples that were flagged by **Ikarus** as part of the *coogos* family have been majorly flagged (80%) by **ESET-NOD32** as belonging to the *kuogo* family. We deduce that **ESET-NOD32**’s *kuogo* is in fact **Ikarus**’s *coogos*. Yet, we are convinced that this label equivalence requires a much deeper analysis to be able to compare equivalent families from different AVs. This phenomenon is even confirmed by the industry [142], which indicated that different AVs give different names to the same family. Using techniques, such as Rand Index, to measure similarity between label clusters of AV families along side label unification is essential so as not to introduce subtle noise in the classifier.

### 7.2. Limitations and Biases

In our study, we have been faced with several limitations, which are:

- Lack of transparency and limited information from AV vendors regarding data on malware families: We were not able to obtain relevant information except from the vendors on which we reported. Hence, the real-world status of malware families might differ from our version.
- Lack of ground-truth for samples in our dataset: While we tried our best to use previously flagged samples, we set our initial threshold at 1 engine before rescanning them on VT. The rescanning yielded a minimum of 5 flaggings per samples and considered 300 K samples as benign. However, we cannot confirm that all of the samples in the rescanned set are malware or that the labels in the VT reports are 100% accurate.
- We lacked knowledge about the flagging influence among the AVs for this particular dataset, especially that it includes only Android samples. While Zhu et al. [140] were able to identify relationships and dependencies between several AV engines, we could not claim that these inter-dependencies extend to our dataset.
- We were unable to study and analyze different generic labels because of the different taxonomies used by each AV. This affected our ability to extract any possible existing FAM/CLASS intelligence by updating AVCLASS2 with new family/class names, which, of course, could have changed the output of the coherence experiments seen in Figure 5.

## 8. Related Work

With the aim of finding a way that allows comparing classification results using various detectors, Maggi et al. [152] studied the malware naming (in)consistencies of four AV engines on a dataset of around 100 K samples. After scanning the samples on VT, they built the taxonomy tree of each AV using patterns from the VT results. They divided the samples into sets based on their assigned values for **class**, **family**, **platform**, and **variant** (for ease, we use AVCLASS’s categorization rather than the authors’). If the mapping of any given set from AV vendors *A* and *B* is one-to-one, a consistency exists. Yet, if samples of one set from *A* match several sets from *B*, then it is a weak inconsistency. Similarly, if the relationship is many-to-many, they consider this a strong inconsistency. They found that overall consistency was better among the variants compared to that of families,which goes against the logical assumption that labels from different AVs would agree more on higher level categories.

Zhu et al. [140] studied a set of 14 K files by querying them on VT for one year and analyzing the results of 65 engines. They measured how individual engines flipped the label of a file over time. They discovered that 50% of the flips were very short-lived (hazard flips) as they tended to flip again the next day. The final result was 1.7 M hazard flips versus 811 K non-hazard ones, and that 64 engines out of 65 had flips. They found that the threshold-based label aggregation, i.e., considered a sample malicious if flagged by more than *t* AVs, is unexpectedly effective in allowing label dynamics only when *t* is given a correct value, unlike the more common threshold of 1 used in many research works. Regarding AV relationships, there were 5 clusters that ranged from the size of 2 to 6 engines with a similar label sequence pattern. Additionally, the authors analyzed the commonly known *reputable engines* from other works, showing that they are not necessarily the most accurate ones.

Kadir et al. [20] discussed the disparity of labels in the context of Android financial malware. They supported their claim by discussing the *Zitmo* family, which was first addressed in Malgenome, and for which engines failed to provide more specific labels even to one sample of that family and instead provided generic labels. The authors made efforts to build a curated dataset of Android financial malware with clear taxonomy and classification after checking the samples themselves to avoid any bias.

Botacin et al. conducted a systematic study to identify the challenges and pitfalls in malware research [25]. The authors reviewed 491 papers from top security conferences between 2000 and 2018. They provided a group of common steps that are must-have for security papers: (1) Research Objective Definition, (2) Hypothesis Definition and Research Requirements, (3) Solutions Design, (4) Experiment Design, and (5) Solution Evaluation. They classified the pitfalls into 20 different categories and identified a set of 5 challenges. The research objectives that they determined based on their analysis of the set of papers were (1) Engineering Solutions, (2) Offensive Techniques, (3) Observational Studies, and (4) Network Traffic. This is slightly different than the set of objectives that we identified though our set is based on a much smaller collection of papers, as well as being more focused on Android.

Zhang et al. [153] proposed a hybrid representation learning technique in which they involve not only the raw labels retrieved from AV engines after querying VT, but also by including other information: static analysis results and app metadata. This work was motivated by the lack of knowledge of how the engines decide on giving a certain label to a specific sample. Their objective was to combat the confusion caused when using label unification tools on samples that have “weak” raw labels. Weak labels maybe generic labels that are immediately discarded by label unification tools or maybe controversial labels in which the majority voting used by the previously mentioned tools becomes less confident. They mentioned that the vendors do not often use CARO and CME conventions. Yet, we showed earlier that even with just a variant in the label, an AV would still be following the CARO standard. However, we agree with the authors of Reference [153] that such labeling is vague and does not allow for reliable analysis. On the other hand, we note that, in our study, we have shown how such disagreement on family name might not even have an impact because of the high similarity in the family clusters for each pair. We are yet to know how true this is in the case of family coherence values for *N* engines collectively; N>2.

Pirch et al. [154] developed a method, called TAGVET, whose objectives is vetting tags from malware collections. The method addresses the fact that most tags are automatically generated, rather than being manually assigned and that sometimes, because of the automation, a misalignment occurs between a tag and the sample’s behavior. TAGVET is based on explainable ML through which it connects tags to the behavioral pattern of a sample after running dynamic analysis. The authors test their method on a Windows-only PHAs set for three tag classes: (1) Sandboxing: based on reports after running samples in Sandbox, (2) Family name extraction: based on output of AVCLASS, and (3) Behavior-based clustering: the technique is based on extracting tuples of system calls and arguments, then using complete-linkage clustering. The accuracy results for the three tag classes ranges between 92% and 97%.

## 9. Conclusions and Future Work

Both the academic and industrial communities rely on the notion of malware family when analyzing emerging or prevalent Android threats, and when working with Android malware datasets. In this work, we explored the usage of malware classification in both communities. In addition, we studied the limitations associated with the use of available malware classification services for determining the family of Android PHAs. This is a critical step for researchers when building labeled datasets as they represent the ground truth when training classifiers. Our analysis of 2.5 M malicious Android samples reveals that the detection coverage of current AV engines is low and that engines often resort to generic family labels. We explored the extent to which different AV engines agree upon flagged samples and their families and found that the level of agreement is quite low, with the best agreement levels being at 52% and 29%, consecutively. The coherence results, especially for family, suggest that the industry has a general agreement on those threats that are well-defined. Based on our study, we were able to provide recommendations for the labeling process (see Section 7.1) to researchers who plan to build their own datasets. While we measured the coverage and family and class coherence for AV pairs, our goal is to extend these experiments to involve more engines. Besides, we believe that the study of the generic labels and the main keywords that engines use as generic ones or to represent generic threats is necessary to deeply understand the basis on which these labels are generated. Moreover, we believe it is important to know the behavior of engines with regard to flagging benign samples as malicious and, thus, would like to study the possible labels that engines would give to those samples. Furthermore, based on our observations of the usage of numbers as potential family names and the interchanging of uppercase and lowercase in the FAM part of the label, we would like to study the automatically generated labels that are related to possibly unseen families and those that are generated because of the engines’ ability to attribute them to previously seen families. Finally, taking into account the lack of consensus, we would like to carry out a study that measures the possible improvement of outputs of label unifying tools by involving real time behavioral monitoring to enhance threat intelligence, based on concepts used in recent studies on relevant Windows malware [154].

## Figures and Tables

**Table 1:** Availability of informative reports that provide top families per AV service of the companies in the table. H1, H2: a report of the first and second half of the year, respectively; Y: company provides a full-year report. The last row represents how many vendors reported on that year, regardless of the number of reports/vendor issued.

Company	Year (20xx)								
	**12**	**13**	**14**	**15**	**16**	**17**	**18**	**19**	**20**
Symantec^®^	-	-	-	Y [60]	Y [61]	Y [62]	Y [63]	-	-
Nokia^®^	-	-	H2 [64]	H1,H2 [65,66]	H1,H2 [67,68]	Y [69]	Y [70]	-	Y [71]
Sophos^®^	-	Y [72]	-	-	Y [73]	Y [74]	-	-	-
Kaspersky^®^	Y [75]	-	Y [76]	Y [77]	Y [78]	Y [79]	Y [80]	Y [81]	Y [82]
CheckPoint^®^	-	-	-	-	H1 [83]	Y [84]	Y [85]	Y [86]	-
Google^®^	-	-	-	-	Y [87]	Y [88]	Y [89]	-	-
Companies/Yr Tot.	1	1	2	3	6	6	5	2	2

**Table 2:** Top common families found in Android threat intelligence reports 2014-2020. Reports column refers to total number of reports in that year. K: Kaspersky, SM: Symantec, N: Nokia, SP: Sophos, C: CheckPoint.

Year	Reports	Common Families
14	2	N/A
15	3	**Opfake** (K, SM), **Lotoor** (K, SM), **Leech** (N, K)
16	6	**Opfake** (SM,SP), **FakeInst** (SM,SP), **HiddenApp** (SM,N),
		**Hummingbad** (C,G), **Ztorg** (C,K), **Rootnik** (SM,N)
17	6	**Simplocker** (SM, SP), **Rootnik** (N, SP), **Sivu** (K, N), **Ztorg** (K, N),
		**Hiddad** (K,C), **Triada** (K,C), **Ztorg** (K,C)
18	5	**Triada** (G, K, N, C), **HiddenApp** (SM, N), **Lotoor** (K, C), **Xiny** (G, N)
19	2	**Necro** (K, C)
20	2	**Hiddad** (N, K)

**Table 3:** Android malware datasets used in security conferences’ papers over the last decade. In # samples column, in case of a repository (repo), we include the range of samples from that repo used by the papers. We use **UNK** whenever it is not possible to identify # samples from that repo (e.g., when several datasets/repos are used collectively, and authors do not provide the # samples used from each of them). The **Summary** row represents the sum of the papers we analyzed and other key information where relevant/easily extracted information can be included, e.g., papers using dataset/year.

Dataset	# Papers	Labl	# Fams	# Samples	VT	Class. Mthd	Collection Time (20xx)	Papers Using the Dataset by Year (20xx)										
								10	11	12	13	14	15	16	17	18	19	20
Malgenome (MG) [10]	34 (42%)	Y	49	1260	N	Manual	10–11			4	4	3	9	6	4	2		1
Contagio (repo) [90]	17 (21%)	N	-	UNK-395	N	N/A	11-			2	2	2	3	2	4	1		1
Drebin [12]	14 (17%)	Y	179	5560	Y	Own	10–12					1		4	7	1		1
VirusShare [91]	10 (12%)	N	-	11 K–35 K	N/A	N/A	N/A					1	1	1	4	1	1	1
VT-malware [92]	9 (11%)	N	-	2 K-238 K	Y	N/A	N/A								4	3	1	
DroidBench [93]	4 (5%)	N	-	UNK-200	N	N/A	N/A						2	1				
AMD [94]	3 (3.7%)	Y	71	24 K	Y	Own	10–16								1	1		2
SandDroid (repo) [95]	3 (3.7%)	N	-	112-38 K	N/A	N/A	N/A					2	1					
Androzoo (repo) [96]	3 (3.7%)	N	-	3 K–13 K	Y	N/A	N/A									1	2	
GPlay-mal (repo) [97]	3 (3.7%)	N	-	UNK-27	Y	N/A	N/A			1					1			1
Andrubis [98]	3 (3.7%)	N	-	422 K	Y	N/A	N/A							1	1			2
DARPA [99]	2 (2.5%)	N	-	11	N	N/A	N/A						1	1				
RmvDroid [23]	1 (1.2%)	Y	56	9.1 K	Y	AVClass	14,15,17										1	
alt markets(repo) [100,101,102,103]	1 (1.2%)	N	-	2 K	Y	N/A	N/A								1			
AndroMalTeam [104]	1 (1.2%)	N	-	34	N	N/A	N/A									1		
Marvin [22]	1 (1.2%)	N	-	15 K	Y	N/A	N/A								1			
Github (repos) [105,106,107,108,109]	1 (1.2%)	N	-	5	Y	N/A	N/A								1			
AndroTotal [110]	1 (1.2%)	N	-	4.1 K	N	N/A	N/A						1					
AndRadar [111]	1 (1.2%)	N	-	N/A	Y	N/A	N/A						1					
CICAndMal17 [112]	1 (1.2%)	N	-	4.3 K	Y	N/A	N/A										1	
Wang et al. [113]	1 (1.2%)	N	-	4.5 M	Y	AVClass	09-17										1	
AndroPUP [114]	1 (1.2%)	N	-	4.6 M	Y	N/A	N/A											1
Spreitzenbarth et al. [115]	1 (1.2%)	N	-	7.5 K	Y	N/A	N/A							1				
MobiSec Lab [116]	1 (1.2%)	N	-	2 K	N/A	N/A	N/A							1				
HackingTeam [117]	1 (1.2%)	N	-	1	N	N/A	N/A							1				
ashishb [118]	1 (1.2%)	N	-	298	N	N/A	N/A									1		
M0Droid [17]	1 (1.2%)	N	-	N/A	Y	N/A	N/A							1				
Canfora et al. [119]	1 (1.2%)	N	-	2	N	N/A	N/A								1			
Sherlock vs. Moriarty [120]	1 (1.2%)	N	-	12	Y	N/A	2016									1		
Companies [121,122,123,124,125,126]	12 (15%)	N	-	69-1.5 K	N/A	N/A	N/A			1	1	3	2	1	4			
Custom [16,21,127,128,129,130,131,132]	9 (11%)	N	-	2-1 K	Y/N	N/A	N/A		2		2		3	1		1		
unknown [133,134,135]	3 (3.7%)	N	-	20–362	Y/N	N/A	N/A				1	1						
**Summary**	**81**	**-**	**49–179**	**UNK-422 K**	**-**	**-**	**10-UNK**	**0**	**2**	**6**	**8**	**9**	**13**	**11**	**17**	**7**	**5**	**4**

**Table 4:** Date ranges for the samples of well-known datasets.

Dataset	First Seen Range
Malgenome	14 October 2009–12 June 2012
Contagio	25 February 2011–20 March 2018
Drebin	14 October 2009–10 August 2013
VirusShare	11 April 2010-UNK
AMD	17 November 2010–14 May 2016

**Table 5:** Objective(s) and main focus of our set of papers.

Research Purpose	# Papers
Detection	34
Analysis/Measurement	23
Tools	21
Family Classification	8
Other	5

**Table 6:** Datasets used in our analysis. The 1st_seen range is according to VT and for the samples we collect (as opposed to the samples in the entire repository, such as in Table 4). For instance, in the case of VirusShare, it relates to the snapshot of 170 K samples. Total refers to total apps used in the experiments after discarding duplicates.

Source	Samples	1st_seen Range
Malgenome	1.2 K	14 October 2009–12 June 2012
Contagio	1.6 K	25 February 2011–20 March 2018
Drebin	5.6 K	14 October 2009–10 August 2013
AMD	24 K	17 November 2010–14 May 2016
Palo Alto	104 K	7 November 2011–3 May 2019
OTX	116 K	26 September 2012–9 July 2020
VirusShare	170 K	11 April 2010–27 December 2019
AndroZoo	2.24 M	14 October 2009–3 November 2020
**Total**	**2.47 M**	**∼late 2009 to late 2020**

## Data Availability

We plan to upload the dataset we used to https://github.com/mra12/labelingDataset.

## Appendix A. Paper Distribution over Conferences

The analysis of papers from mentioned conferences that we discussed in Section 3.2 are found in Table A1.

**Table A1:** Distribution of papers where a dataset with Android malware was used with respect to conferences and years.

Venue	Year (20xx)											Total
	10	11	12	13	14	15	16	17	18	19	20	
CCS	-	1	1	2	2	1	1	1	1	1	1	11
USENIX	-	-	2	-	-	-	-	2	2	1	-	7
RAID	-	-	-	1		2	1	1	-	2	-	7
ACSAC	-	1	1	1	2	2	1	4	3	-	1	16
DIMVA	-	-	2	-	1	1	-	1	-	-	-	5
ASIACSS	-	-	-	2	1	3	4	5	-	1	1	17
S&P	-	-	-	-	-	2	1	-	-	-	-	3
NDSS	-	-	-	2	3	2	3	3	1	-	1	15
**sum Yr**	**-**	**2**	**6**	**8**	**9**	**13**	**11**	**17**	**7**	**5**	**4**	**81**
